# The relationship between obesity and patient-reported outcome measures in people with polymyalgia rheumatica

**DOI:** 10.1093/rap/rkae081

**Published:** 2024-07-05

**Authors:** Ian C Scott, Ram Bajpai, Samantha L Hider, Toby Helliwell, Christian D Mallen, Sara Muller

**Affiliations:** Primary Care Centre Versus Arthritis, School of Medicine, Keele University, Keele, UK; Haywood Academic Rheumatology Centre, Haywood Hospital, Midlands Partnership University NHS Foundation Trust, Staffordshire, UK; Primary Care Centre Versus Arthritis, School of Medicine, Keele University, Keele, UK; Primary Care Centre Versus Arthritis, School of Medicine, Keele University, Keele, UK; Haywood Academic Rheumatology Centre, Haywood Hospital, Midlands Partnership University NHS Foundation Trust, Staffordshire, UK; Primary Care Centre Versus Arthritis, School of Medicine, Keele University, Keele, UK; Centre for Academic Social Care, Public Health, Community and Primary Care (COSMIC), Midlands Partnership University NHS Foundation Trust, Staffordshire, UK; Primary Care Centre Versus Arthritis, School of Medicine, Keele University, Keele, UK; Haywood Academic Rheumatology Centre, Haywood Hospital, Midlands Partnership University NHS Foundation Trust, Staffordshire, UK; Primary Care Centre Versus Arthritis, School of Medicine, Keele University, Keele, UK

**Keywords:** PMR, BMI, obesity, patient-reported outcome measures

## Abstract

**Objective:**

To examine the association between obesity and patient-reported outcome measures (PROMs) in a primary care-based cohort of people with PMR.

**Methods:**

The PMR Cohort Study recruited people with incident PMR from 382 general practices. Self-completed questionnaires (0, 12, 24 months) captured a range of PROMs for pain, stiffness, anxiety, depression, fatigue, function and quality of life, alongside data on BMI. People were categorized as underweight/normal weight (BMI < 25kg/m^2^), overweight (25–29.99 kg/m^2^) or obese (≥30 kg/m^2^). Piecewise, multilevel, linear mixed-effects regression models examined relationships between BMI categories and PROMs over time, adjusting for confounding variables. Chi-squared tests examined the relationship between obesity and glucocorticoid persistence.

**Results:**

644 people with PMR were included. At baseline, 33.9% were normal/underweight, 40.6% overweight and 25.5% obese. Compared with normal/underweight people, those with obesity had significantly worse scores for the following: pain and stiffness at 12 months; fatigue at 12 and 24 months; depression at baseline; physical function at all time points; and quality of life at baseline and 12 months. They also had significantly smaller improvements in stiffness (1.13 units on an 11-point numeric rating scale; *P *=* *0.001) and physical function (0.14 units measured using the modified Health Assessment Questionnaire; *P *=* *0.025) between 0 and 12 months. BMI categories did not relate to persistent glucocorticoid use at 12 months (*P *=* *0.110) or 24 months (*P *=* *0.166).

**Conclusion:**

Obesity associates with poorer outcomes for a range of PROMs in people with PMR. Consideration should be given to providing weight management support to people with PMR and obesity.

Key messages

- People with PMR and obesity have worse scores for many patient-reported outcome measures over time.
- Obesity associates with significantly smaller 12-month improvements in stiffness and function following PMR diagnosis.

## Introduction

Obesity is a major global health issue, affecting >1 billion people worldwide [1]. England has one of the highest rates of obesity in Europe, with approximately two-thirds of adults overweight/obese [2]. As obesity predisposes to a pro-inflammatory state through increased interleukin-6 and tumour necrosis factor-α production, and reduced levels of anti-inflammatory adiponectin [3], previous studies have examined its role in the development and perpetuation of inflammatory arthritis, reporting that people who are obese are more likely to develop RA [4] and less likely to achieve RA remission [5]. Similarly, cohort studies demonstrate dose-dependent associations between obesity and PsA development [6, 7] and lower response rates to tumour necrosis factor-inhibitors in people with axial spondyloarthritis that are obese [8].

Similar to inflammatory arthritis, PMR is a common, immune-driven condition, characterized by inflammation and raised interleukin-6 levels [9]. It is, therefore, reasonable to hypothesize that obesity could also play an important role in its development and progression. To date, few studies have examined this. Cimmino *et al.* [10] reported that amongst 83 people with incident PMR, obesity (BMI ≥30 kg/m^2^) was associated with higher pain and fatigue scores and worse function at diagnosis, and higher levels of glucocorticoid use over 12 months. Hoganson *et al.* [11] reported no association between a ‘high’ BMI (≥25kg/m^2^) and PMR development or duration of glucocorticoid use amongst 364 people with PMR and 364 controls. Esen *et al.* [12] reported no relationship between baseline obesity and achieving steroid-free remission in a small cohort of 50 people with PMR. In view of these contrasting findings, we examined the association between obesity and patient outcomes in people with incident PMR. Specific objectives were to establish if obesity associates with: (1) worse patient-reported outcome measure (PROM) scores over time, (2) smaller improvements in PROM scores over time and (3) higher levels of persistent glucocorticoid use.

## Methods

### Subjects

We evaluated data from people recruited to the PMR Cohort Study, a primary care-based inception cohort enrolling from 382 general practices between 2012 and 2014 [13]. Described previously, people with GP-diagnosed incident PMR were recruited at diagnosis [14]. Participants were mailed a baseline questionnaire, with all responders mailed further questionnaires at 1, 4, 8, 12, 18 and 24 months (unless withdrawing). Enrolment did not affect usual care. The current analysis is restricted to the 644 people (from 652 recruited) aged ≥50 years, as this age cut-off is a universal PMR classification criteria component [15].

### Ethics

Ethical approval for the PMR Cohort study was received from the Staffordshire Research Ethics Committee (REC reference number: 12/WM/0021). All recruited people provided written informed consent.

### Sociodemographic characteristics and glucocorticoid use

Baseline questionnaires captured sociodemographic data. Age and gender were available in the GP study referral form. Height, weight and glucocorticoid use were self-reported at baseline, and weight and glucocorticoid use at 12 and 24 months.

### Patient-reported outcome measures

The following were evaluated at baseline, 12 and 24 months: (a) PMR-related pain and stiffness using 0–10 numeric rating scales (NRS); (b) anxiety using the Generalized Anxiety Disorders (GAD-7) Questionnaire (scoring 0–21; scores of 5, 10 and 15 proposed cut-points for mild, moderate and severe anxiety, respectively) [16]; (c) depression using the Patient Health Questionnaire (PHQ-8) (scoring 0–24; scores ≥10 defining current depression) [17]; (d) fatigue using the 13-item Functional Assessment of Chronic Illness Therapy—Fatigue (FACIT-Fatigue) questionnaire [18] (scoring 0–52; higher scores indicating less fatigue); (e) function using the modified Health Assessment Questionnaire (mHAQ) (scoring 0–3; higher scores indicating more disability) [19]; (f) quality of life using the EQ-5D-3L (scoring from <0 [worse than death] to 1 [perfect health]) [20].

### Obesity

Obesity was defined using National Institute for Health and Care Excellence BMI categories of underweight (<18.5 kg/m^2^), normal weight (18.5–24.9 kg/m^2^), overweight (25–29.9 kg/m^2^) and obese (≥30 kg/m^2^) [21]. There were three participants at baseline whose BMI was just below the normal threshold (ranging 17.97–18.38 kg/m^2^). These participants were merged with the normal category and presented together to avoid any information loss.

### Statistical analysis

#### Summary statistics

Descriptive statistics (mean [s.d.], or median with interquartile range [IQR]) for continuous variables; proportions for categorical variables described baseline participant characteristics and PROMs scores, alongside glucocorticoid use (yes/no) at 0, 12 and 24 months.

#### Linear mixed-effects models

Piecewise linear mixed-effects regression models evaluated relationships between BMI categories and PROMs, adjusting for relevant covariates. Restricted maximum-likelihood estimation and robust variance were used to estimate the average treatment effect across two follow-up time points (12 and 24 months) in a repeated measures design. Linear mixed-effects models account for the correlation between repeated measurements within individuals, improving parameter estimation accuracy. Moreover, they can handle missing outcome data, allowing inclusion of all available outcome data in the analysis, reducing bias and optimizing power [22]. Separate models were constructed for each PROM, with the outcome of interest included as the response variable. Each model included BMI category, time and a BMI category*time interaction term as explanatory variables, alongside the potential confounding variables age (at diagnosis), gender, glucocorticoid (prednisolone) dose (at each time point), smoking status (at baseline) and alcohol intake (at baseline) [23]. As multiple imputation is recommended in longitudinal data analysis with missing covariates [22], sensitivity analyses imputed missing baseline covariates and missing outcomes at any follow-up time using multiple imputation techniques with 20 imputations (based on the missingness pattern) following Rubin’s rule [24]. The association between BMI categories and glucocorticoid use at 12 and 24 months was evaluated using Chi-squared tests. *P*-values <0.05 were considered statistically significant.

#### Software

Analyses were conducted using Stata version 18.0 (StataCorp, Lakeway Drive, College Station, Texas, USA).

## Results

### Baseline patient characteristics

Of the 644 people with PMR included in the analysis (Table 1), their mean age was 72.8 (s.d. 8.6) years, and the majority were female (61.7%) and of white ethnicity (97.8%). BMI in study participants at baseline ranged from 17.97 to 57.47 kg/m^2^, with 40.6% categorized overweight and 25.5% obese. BMI scores changed little over follow-up (Supplementary Fig. S1, available at *Rheumatology Advances in Practice* online).

**Table 1.** Baseline demographic, clinical and outcome characteristics of study participants

Characteristic	Summary statistic
*Sociodemographic*	
Age, mean (s.d.), years	72.8 (8.6)
Female gender, *n* (%)	397 (61.7)
White ethnic group, *n* (%)	630 (97.8)
Smoking status, *n* (%) [*n* = 635]	
Never	312 (49.1)
Previous	283 (44.6)
Current	40 (6.3)
Alcohol consumption, *n* (%) [*n* = 642]	
Daily	82 (12.8)
3–4 times a week	81 (12.6)
1–2 times a week	111 (17.3)
1–3 times a month	79 (12.3)
Special occasions	179 (27.9)
Never	110 (17.1)
BMI categories, *n* (%) [*n* = 614]	
Normal/underweight (<25 kg/m^2^)	208 (33.9)
Overweight (25–29.9 kg/m^2^)	249 (40.6)
Obese (>30 kg/m^2^)	157 (25.5)
BMI in kg/m^2^, median (IQR) [*n* = 614]	26.6 (24.1 to 30.1)
*PMR treatment and PMR-related pain and stiffness*	
Current use of prednisolone, *n* (%) [*n* = 636]	620 (97.5)
Current daily prednisolone dose in mg, median (IQR) [*n* = 530]	15.0 (12.5, 20.0)
PMR-related pain: numeric rating scale, median (IQR) [*n* = 632]	8.0 (7.0, 9.0)
PMR-related stiffness: numeric rating scale, median (IQR) [*n* = 634]	8.0 (7.0, 9.0)
*Other patient-reported outcome measures*	
Depression: PHQ-8, median (IQR) [*n* = 593]	4.0 (1.0, 8.0)
Anxiety: GAD-7, median (IQR) [*n* = 601]	2.0 (0.0, 6.0)
Fatigue: FACIT-fatigue, median (IQR) [*n* = 618]	36.7 (24.8, 44.0)
Function: mHAQ, median (IQR) [*n* = 620]	0.4 (0.0, 1.0)
Quality of life: EQ-5D-3L, median (IQR) [*n* = 594]	0.7 (0.6, 0.9)

Cohort size is 644 patients. Current prednisolone dose is in those reporting current prednisolone use.

The number of patients with complete data for each variable are provided in square brackets.

PHQ: Patient Health Questionnaire; GAD: Generalized Anxiety Disorders Questionnaire; mHAQ: Modified HAQ; FACIT-F: Functional Assessment of Chronic Illness Therapy—Fatigue; EQ-5D-3L: EuroQol five-dimension scale.

### Baseline PROM scores

Median PMR-related pain and stiffness scores were both 8.0 (IQR 7.0, 9.0). Median PHQ-8 scores of 4.0 (IQR 1.0, 8.0) indicated many had mild depression [25], and median GAD-7 scores of 2.0 (IQR 0.0, 6.0) suggested most did not experience clinically relevant anxiety [16]. Median FACIT-Fatigue scores of 36.7 (IQR 24.8, 44.0) were below normal population levels (mean 43.5) [26] indicating greater levels of fatigue; median mHAQ scores of 0.4 (IQR 0.0, 1.0) indicated generally low levels of disability and EQ-5D-3L scores of 0.7 (IQR 0.6, 0.9) were slightly below the English population normal value of 0.86 [27] indicating worse quality of life.

### Relationship between obesity and PMR-related pain and stiffness

**Figure 1.**
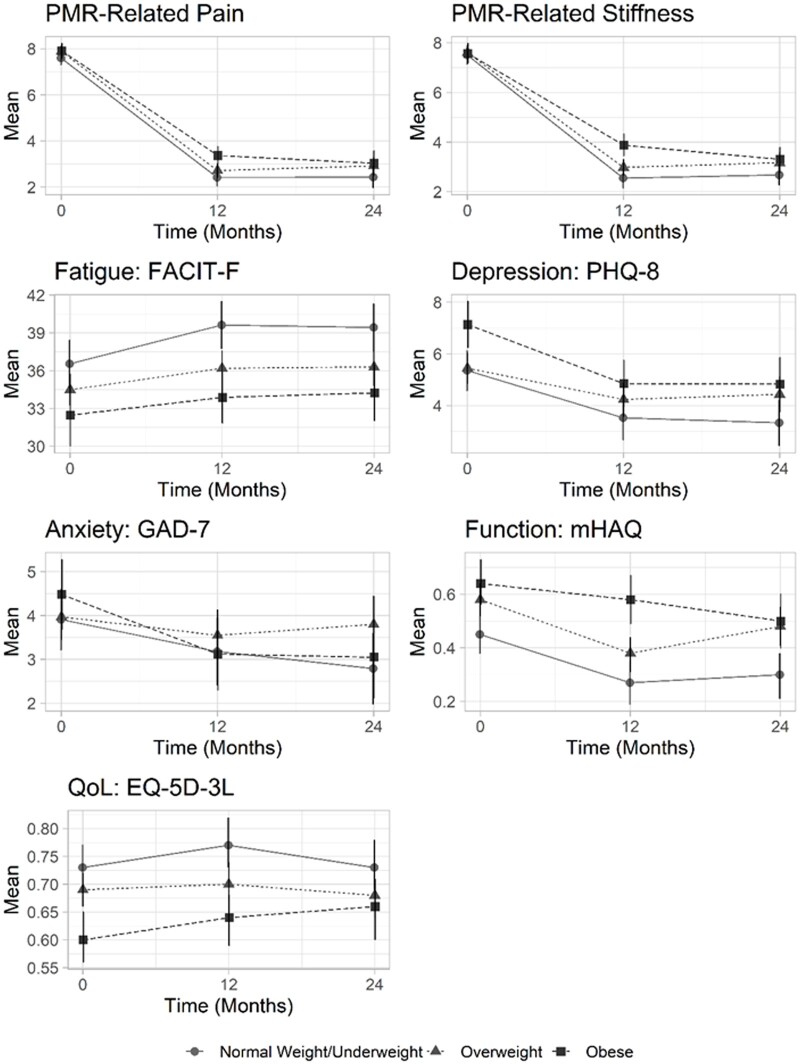
Predicted mean PROM scores in people with PMR that are obese, overweight and normal weight/underweight. Predicted mean outcome scores with 95% CI bars from the mixed-effects models are plotted for each outcome at each time-point in people categorized as obese, overweight and normal weight/underweight. PMR-related pain and stiffness are measured on 11-point numeric rating scales. PHQ-8: 8-item Patient Health Questionnaire; GAD-7: 7-item Generalized Anxiety Disorders Questionnaire; mHAQ: Modified Health Assessment Questionnaire; FACIT-Fatigue: Functional Assessment of Chronic Illness Therapy—Fatigue questionnaire; EQ-5D-3L: EuroQol five-dimension scale

At all time points, predicted mean pain and stiffness scores were numerically higher in patients categorized as overweight or obese compared with those categorized as normal weight/underweight (Table 2; Fig. 1). In the mixed-effects models, these differences were only significantly different for those categorized as obese *vs* normal/underweight for pain at 12 months (mean 3.37 [95% CI 2.97, 3.76] *vs* 2.42 [2.06, 2.78]) and stiffness at 12 months (mean 3.87 [3.45, 4.32] *vs* 2.55 [2.15, 2.95]).

**Table 2.** PROM scores and their associations with obesity using linear mixed-effects models

Outcome measures	Normal weight/underweight (BMI <25)	Overweight (BMI 25.0-29.9)	Obese (BMI >30)	Overweight *vs* normal weight/underweight		Obese *vs* normal weight/underweight	
	Mean (95% CI)	Mean (95% CI)	Mean (95% CI)	MD (95% CI)	*P*-value	MD (95% CI)	*P*-value
PMR-related pain (numeric rating scale)							
Baseline	7.60 (7.31, 7.89)	7.84 (7.58, 8.1)	7.91 (7.57, 8.24)				
12 months	2.42 (2.06, 2.78)	2.66 (2.34, 2.97)	3.37 (2.97, 3.76)	0.00 (−0.59, 0.59)	0.993	0.64 (−0.02, 1.30)	0.056
24 months	2.43 (1.96, 2.91)	2.90 (2.48, 3.32)	3.04 (2.51, 3.58)	0.23 (−0.48, 0.94)	0.522	0.31 (−0.50, 1.10)	0.456
PMR-related stiffness (numeric rating scale)							
Baseline	7.51 (7.15, 7.87)	7.62 (7.30, 7.94)	7.57 (7.16, 7.98)				
12 months	2.55 (2.15, 2.95)	2.92 (2.57, 3.27)	3.87 (3.45, 4.32)	0.19 (−0.41, 0.79)	0.533	1.13 (0.46, 1.80)	**0.001**
24 months	2.68 (2.26, 3.10)	3.21 (2.83, 3.57)	3.31 (2.83, 3.79)	0.35 (−0.38, 1.08)	0.347	0.64 (−0.19, 1.47)	0.131
Fatigue (FACIT-fatigue)							
Baseline	36.54 (34.63, 38.44)	35.18 (33.38, 36.99)	32.48 (30.02, 34.93)				
12 months	39.62 (37.74, 41.50)	36.97 (35.34, 38.61)	33.88 (31.87, 35.88)	0.42 (−1.80, 2.65)	0.709	−0.84 (−3.39, 1.72)	0.521
24 months	39.43 (37.52, 41.33)	36.94 (35.22, 38.66)	34.24 (32.02, 36.46)	0.21 (−2.29, 2.71)	0.871	−1.40 (−4.29, 1.50)	0.345
Depression (PHQ-8)							
Baseline	5.36 (4.59, 6.12)	5.45 (4.78, 6.12)	7.13 (6.23, 8.03)				
12 months	3.53 (2.68, 4.37)	4.13 (3.37, 4.89)	4.85 (3.95, 5.76)	0.32 (−0.68, 1.32)	0.532	−0.01 (−1.13, 1.11)	0.985
24 months	3.34 (2.44, 4.23)	4.04 (3.25, 4.84)	4.83 (3.80, 5.86)	0.71 (−0.46, 1.88)	0.236	0.18 (−1.17, 1.52)	0.798
Anxiety (GAD-7)							
Baseline	3.91 (3.22, 4.60)	3.88 (3.25, 5.50)	4.48 (3.68, 5.28)				
12 months	3.18 (2.41, 3.95)	3.32 (2.64, 4.01)	3.12 (2.30, 3.95)	0.12 (−0.76, 1.01)	0.783	−0.41 (−1.40, 0.57)	0.410
24 months	2.79 (1.98, 3.60)	3.13 (2.42, 3.84)	3.05 (2.12, 3.97)	0.86 (−0.22, 1.93)	0.118	0.26 (−0.96, 1.48)	0.675
Function (mHAQ)							
Baseline	0.45 (0.38, 0.53)	0.57 (0.50, 0.63)	0.64 (0.56, 0.73)				
12 months	0.27 (0.19, 0.35)	0.37 (0.30, 0.44)	0.58 (0.49, 0.67)	−0.02 (−0.12, 0.09)	0.764	0.14 (0.02, 0.26)	**0.025**
24 months	0.30 (0.21, 0.38)	0.48 (0.40, 0.55)	0.50 (0.40, 0.60)	0.04 (−0.08, 0.16)	0.519	0.04 (−0.10, 0.18)	0.546
Quality of Life (EQ-5D-3L)							
Baseline	0.73 (0.69, 0.77)	0.69 (0.65, 0.72)	0.60 (0.56, 0.65)				
12 months	0.77 (0.73, 0.82)	0.70 (0.65, 0.74)	0.64 (0.59, 0.68)	0.00 (−0.06, 0.06)	0.941	−0.02 (−0.08, 0.05)	0.602
24 months	0.73 (0.69, 0.78)	0.68 (0.64, 0.72)	0.66 (0.60, 0.71)	0.03 (−0.04, 0.09)	0.434	0.04 (−0.03, 0.12)	0.253

Adjusted mean scores and MDs with 95% CIs and *P*-values from linear mixed-effects regression models that include the explanatory variables BMI category, time, BMI category * time interaction term, age, gender, prednisolone dose, smoking status and alcohol intake. *P*-values <0.05 highlighted in bold.

Reductions in pain and stiffness were largely confined to the first 12 months for all BMI categories (Fig. 1; Supplementary Table S1, available at *Rheumatology Advances in Practice* online), with numerically smaller reductions seen for obese *vs* normal/underweight people for pain at 0 to 12 months (mean change −4.43 [−4.94, −3.92] *vs* −5.07 [−5.52, −4.62]) and stiffness at 0 to 12 months (mean change −3.74 [−4.26, −3.21] *vs* −4.87 [−5.32, −4.41]). In the linear mixed-effects model, this lesser improvement was statistically significant for stiffness at 0 to 12 months (Table 2; mean difference (MD) for obese *vs* normal weight/underweight of 1.13 [0.46, 1.80]; *P *=* *0.001). Sensitivity analyses using imputed missing data did not alter these associations (Supplementary Table S2, available at *Rheumatology Advances in Practice* online).

### Relationship between obesity and other PROM scores

At all time points, except for anxiety, predicted mean scores for all other PROMs were numerically worse in patients categorized as overweight or obese compared with those categorized as normal weight/underweight (Table 2; Fig. 1). In the mixed-effects models, these differences were only significant between those with obesity *vs* normal/underweight for fatigue at 12 months (mean FACIT-fatigue 33.88 [31.87, 35.88] *vs* 39.62 [37.74, 41.50]) and 24 months (mean FACIT-fatigue 34.24 [32.02, 36.46] *vs* 39.43 [37.52, 41.33]), depression at baseline (mean PHQ-8 7.13 [6.23, 8.03] *vs* 5.36 [4.59, 6.12]), function at baseline (mean mHAQ 0.64 [0.56, 0.73] *vs* 0.45 [0.38, 0.53]), 12 months (mean mHAQ 0.58 [0.49, 0.67] *vs* 0.27 [0.19, 0.35]) and 24 months (mean mHAQ 0.50 [0.40, 0.60] *vs* 0.30 [0.21, 0.38]), and quality of life at baseline (mean EQ-5D-3L 0.60 [0.56, 0.65] *vs* 0.73 [0.69, 0.77]) and 12 months (mean EQ-5D-3L 0.64 [0.59, 0.68] *vs* 0.77 [0.73, 0.82]).

As with pain and stiffness, improvements in PROMs in all BMI categories were largely confined to the first 12 months (Fig. 1; Supplementary Table S1, available at *Rheumatology Advances in Practice* online). Significant differences in changes in PROMs over time by obesity category were only seen for mHAQ between 0 and 12 months, which improved less in people that were obese compared with those that were normal weight/underweight (Table 2; MD 0.14 [0.02, 0.26]; *P *=* *0.025). Sensitivity analyses with imputed missing data did not substantially alter these associations (Supplementary Table S2, available at *Rheumatology Advances in Practice* online).

### Relationship between obesity and glucocorticoid use

At baseline, nearly all people with PMR (98.8%) received prednisolone, with a median dose of 15 mg (IQR 10–20 mg) across all BMI groups (Table 1). Over three quarters (80.9%) and more than half (58.3%) continued to receive prednisolone at 12 and 24 months. The proportion receiving glucocorticoids was similar across BMI categories, with no relationship between ongoing prednisolone use and obesity at 12 (*P *=* *0.110) and 24 (*P *=* *0.166) months. It is noteworthy, however, that glucocorticoid use data were missing in 24.2% and 32.6% of people at 12 and 24 months, respectively.

## Discussion

Our study—which involved an in-depth evaluation of the relationship between obesity and a range of PROMs in people with incident PMR managed in primary care—has demonstrated that people with obesity have numerically worse scores for most PROMs at all time points than people that are normal weight/underweight, with these differences being statistically significant for numerous PROMs at various time points. It also shows that people with incident PMR who are obese have smaller improvement in stiffness and function over the first 12 months of care than those that are normal weight/underweight, with differences that are statistically significant after accounting for potential confounding variables. Whilst these findings indicate that obesity is associated with worse outcomes in people with PMR, it is important to note that any differences between BMI categories are small (with reductions in stiffness and mHAQ during the first 12 months of care being 1.13 and 0.14 units less in people that are obese) limiting their clinical relevance.

There is substantial evidence from systematic reviews with meta-analyses that obesity associates with a significantly increased risk of chronic pain [28], depression [29], anxiety [30] and disability [31]. In people with inflammatory arthritis, systematic reviews also demonstrate that obesity is associated with a reduced likelihood of achieving remission/minimal disease activity [5, 32] and responding to anti-TNF therapy [33]. The reasons for these associations are uncertain, and likely involve a broad range of different interacting/additive mechanisms. For example, a systematic review of studies exploring biopsychosocial variables associated with the relationship between obesity and depression reported consistent associations for the severity of obesity, educational attainment, body image, psychological factors, physical health, psychological characteristics, interpersonal effectiveness, binge eating and experience of stigma [34]. In cross-sectional studies using mediation analyses, it has been proposed that leptin (an adipokine mainly secreted by adipose tissue, with proinflammatory effects) partially mediates the association between obesity and pain in hand osteoarthritis [35], that pain is a significant mediator (of 22–44%) of the association between obesity and disability in women aged 70 years and older [36], and that long-term conditions play a mediating role in the relationship between obesity and reduced quality of life [37]. Regardless of the causal pathways, the multiple benefits of optimizing weight, which include reducing the risk of developing diabetes mellitus, improved triglyceride and hypertension levels and reduced levels of hepatic steatosis and hospitalizations [38], suggest that weight loss in people with PMR that are obese should form a part of the care they receive.

Our findings that stiffness improved significantly less over the first 12 months of treatment and that fatigue levels are significantly worse at 12 and 24 months in people with PMR who are obese compared with those who are of normal weight/underweight appear to be of particular importance to people with PMR. A previous survey of 415 people with PMR that sought to explore their priorities for PMR care and research found that 75% identified ‘managing stiffness’ as a priority clinical area, and 78% selected ‘pain, stiffness and fatigue’ as a priority for future research [39]. A qualitative study involving focus groups including 50 people with PMR reported that patients often feel that (unlike with pain) stiffness and fatigue responses to medicines, including steroids, are variable. Our results suggest that obesity may contribute to this perception amongst patients. Whilst it is possible that obesity could lead to lesser improvements in these PROMs due to an increase in body weight meaning a larger dose of glucocorticoids is required compared with normal-weight individuals, the few studies examining the impact of obesity on glucocorticoid pharmacokinetics lack conclusive findings [40], and there are concerns that owing to changes in lean: adipose weight proportions in people with obesity, basing maintenance drug doses on total body weight is unlikely to result in a comparable drug response across different body sizes [41].

Our study’s main strength is that its primary care setting (which is where the majority of people with PMR receive care in the UK) ensures its generalizability. In the UK, the GP has a ‘gatekeeper’ role, meaning that the full spectrum of those diagnosed with PMR was eligible to be included in our cohort, regardless of whether they were referred to a rheumatologist (although whether the care received by the participants in this study is generalizable to patients outside the UK is less clear, as is the case for any study recruiting in a single country). Its additional strengths comprise the inclusion of people at their diagnosis in primary care means that we were able to examine the associations between obesity and PROMs throughout the first two years of the disease and the use of short questionnaires with a reminder system optimized survey response rates (90% at baseline, 82% at 12 months, and 78% at 24 months). Its limitations include the capture of BMI and PROMs data from a limited number of time points over the 2 years, which limits the power of our mixed-effects models and means any short-term variability in PROMs (particularly pain) will not be captured; the use of self-reported questionnaires to ascertain BMI; high levels of missing data for glucocorticoid use and a lack of information on glucocorticoid dose, laboratory outcomes (erythrocyte sedimentation rate and C-reactive protein levels) and potentially important confounding variables (particularly comorbidities). Whilst permission was obtained to link survey responses to participants’ primary care medical records, which would have enabled ascertainment of prescription and laboratory information as well as comorbid diagnoses, changes to the health care landscape during the study period alongside the large number of practices involved meant this was not feasible.

In conclusion, our study shows that people with incident PMR managed in primary care who are obese have worse scores in a range of PROMs over the first 2 years of their care and have significantly smaller improvements in stiffness and function over the first 12 months. This suggests that—when considered alongside the numerous other health benefits—optimizing weight should form part of routine care in people with PMR.

## Supplementary Material

rkae081_Supplementary_Data

## Data Availability

Keele University is a member of the UK Reproducibility Network and committed to the principles of the UK Concordat on Open Research Data. The School of Medicine and Keele Clinical Trials Unit have a longstanding commitment to sharing data from our studies to improve research reproducibility and to maximize benefits for patients, the wider public and the health and care system. We encourage collaboration with those who collected the data, to recognize and credit their contributions. The School of Medicine and Keele Clinical Trials Unit make data available to bona-fide researchers upon reasonable request via open or restricted access through a strict controlled access procedure. The release of data may be subject to a data use agreement (DUA) between the Sponsor and the third party requesting the data. In the first instance, data requests and enquiries should be directed to: medicine.datasharing@keele.ac.uk.
