# Supplementary material for: The relationship between obesity and patient-reported outcome measures in people with polymyalgia rheumatica
Source: Rheumatol Adv Pract. 2024 Jul 5;8(3):rkae081. doi: 10.1093/rap/rkae081 (PMC11239789; doi:10.1093/rap/rkae081)
Supplement: rkae081_Supplementary_Data [file rkae081_supplementary_data.docx]

**Supplementary Figure S1. BMI Scores Over Time.**

**
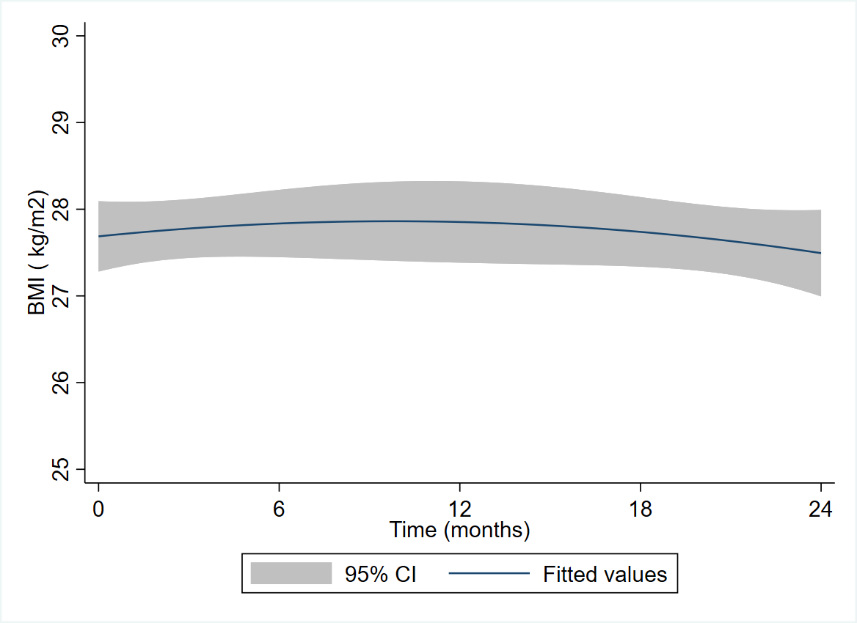
**

**Supplementary Table S1. Mean Changes in PROM Scores in Each Obesity Group Between Time-Points**

| **Outcomes** | **Normal/ under- weight (BMI <25)** | **Overweight**  **(BMI 25.0-29.9)** | **Obese**  **(BMI >30)** |
| --- | --- | --- | --- |
| **PMR-Related Pain (Numeric Rating Scale)** | |  |  |
| 0-12 months | -5.07 (-5.52, -4.62) | -5.08 (-5.49, -4.66) | -4.43 (-4.94, -3.92) |
| 12-24 months | 0.09 (-0.41, 0.60) | 0.33 (-0.13, 0.78) | -0.24 (-0.80, 0.32) |
| **PMR-Related Stiffness (Numeric Rating Scale)** | |  |  |
| 0-12 months | -4.87 (-5.32, -4.41) | -4.68 (-5.10, -4.25) | -3.74 (-4.26, -3.21) |
| 12-24 months | 0.18 (-0.34, 0.69) | 0.33 (-0.13, 0.80) | -0.32 (-0.89, 0.25) |
| **Fatigue (FACIT-Fatigue)** |  |  |  |
| 0-12 months | 1.68 (0.09, 3.26) | 2.10 (0.58, 3.63) | 0.84 (-1.20, 2.88) |
| 12-24 months | 0.14 (-1.40, 1.67) | -0.08 (-1.49, 1.33) | -0.42 (-2.14, 1.29) |
| **Depression (PHQ-8)** |  |  |  |
| 0-12 months | -1.08 (-1.83, -0.34) | -0.76 (-1.47, 0.27) | -1.09 (-2.00, -0.19) |
| 12-24 months | -0.20 (-0.99, 0.59) | 0.19 (-0.53, 0.90) | -0.02 (-0.88, 0.85) |
| **Anxiety (GAD-7)** |  |  |  |
| 0-12 months | -0.29 (-0.94, 0.37) | -0.16 (-0.80, 0.47) | -0.70 (-1.31, 0.62) |
| 12-24 months | -0.55 (-1.25, 0.15) | 0.19 (-0.45, 0.82) | 0.13 (-0.64, 0.89) |
| **Function (mHAQ)** |  |  |  |
| 0-12 months | -0.15 (-0.23, -0.07) | -0.16 (-0.24, -0.09) | -0.01 (-0.11, 0.09) |
| 12-24 months | 0.05 (-0.04, 0.13) | 0.10 (0.03, 0.18) | -0.05 (-0.14, 0.05) |
| **Quality of Life (EQ-5D-3L)** |  |  |  |
| 0-12 months | 0.01 (-0.03, 0.06) | 0.01 (-0.03, 0.06) | -0.01 (-0.06, 0.05) |
| 12-24 months | -0.05 (-0.09, -0.001) | -0.02 (-0.07, 0.02) | 0.01 (-0.04, 0.06) |

Values are mean difference (95% confidence interval).

**Supplementary Table S2. Estimated PROM scores and their association with obesity using linear mixed models at each timepoint after multiple imputation**

| **Outcome measures** | **Normal/ under- weight (BMI <25)** | **Overweight (BMI 25.0-29.9)** | **Obese (BMI >30)** | **Overweight vs Normal** | | **Obese vs Normal** | |
| --- | --- | --- | --- | --- | --- | --- | --- |
|  | **Mean (95% CI)** | **Mean (95% CI)** | **Mean (95% CI)** | **MD (95% CI)** | ***P*-value** | **MD (95% CI)** | ***P*-value** |
| **PMR-Related Pain (Numeric Rating Scale)** | |  |  |  |  |  |  |
| Baseline | 7.63 (7.36, 7.89) | 7.90 (7.66, 8.14) | 7.99 (7.69, 8.30) |  |  |  |  |
| 12 months | 2.59 (2.23, 2.95) | 2.80 (2.48, 3.11) | 3.52 (3.15, 3.89) | -0.07 (-0.62, 0.48) | 0.803 | 0.56 (-0.05, 1.17) | 0.071 |
| 24 months | 2.72 (2.27, 3.16) | 3.10 (2.72, 3.48) | 3.32 (2.87, 3.78) | 0.11 (-0.53, 0.75) | 0.739 | 0.24 (-0.49, 0.97) | 0.523 |
| **PMR-Related Stiffness (Numeric Rating Scale)** | |  |  |  |  |  |  |
| Baseline | 7.55 (7.28, 7.83) | 7.62 (7.37, 7.88) | 7.67 (7.35, 7.98) |  |  |  |  |
| 12 months | 2.71 (2.34, 3.08) | 3.01 (2.69, 3.32) | 3.87 (3.50, 4.23) | 0.22 (-0.32, 0.77) | 0.418 | 1.05 (0.43, 1.67) | **0.001** |
| 24 months | 3.03 (2.54, 3.51) | 3.36 (2.96, 3.75) | 3.73 (3.22, 4.24) | 0.26 (-0.44, 0.96) | 0.463 | 0.60 (-0.19, 1.38) | 0.138 |
| **Fatigue (FACIT-Fatigue)** |  |  |  |  |  |  |  |
| Baseline | 34.77 (33.41, 36.12) | 34.59 (33.38, 35.80) | 32.44 (30.84, 34.04) |  |  |  |  |
| 12 months | 37.52 (36.06, 38.99) | 36.01 (34.69, 37.32) | 33.97 (32.38, 35.56) | -1.34 (-3.21, 0.52) | 0.158 | -1.23 (-3.45, 0.99) | 0.275 |
| 24 months | 37.15 (35.57, 38.73) | 35.89 (34.53, 37.24) | 33.20 (31.27, 35.12) | -1.09 (-3.12, 0.95) | 0.295 | -1.63 (-4.12, 0.86) | 0.199 |
| **Depression (PHQ-8)** |  |  |  |  |  |  |  |
| Baseline | 5.68 (5.05, 6.31) | 5.41 (4.82, 5.96) | 6.33 (5.60, 7.06) |  |  |  |  |
| 12 months | 4.21 (3.50, 4.92) | 4.43 (3.82, 5.05) | 5.22 (4.50, 5.95) | 0.49 (-0.40, 1.38) | 0.281 | 0.36 (-0.69, 1.41) | 0.501 |
| 24 months | 4.03 (3.27, 4.78) | 4.61 (3.98, 5.23) | 5.62 (4.81, 6.43) | 0.85 (-0.10, 1.80) | 0.079 | 0.95 (-0.16, 2.06) | 0.094 |
| **Anxiety (GAD-7)** |  |  |  |  |  |  |  |
| Baseline | 4.19 (3.61, 4.77) | 3.94 (3.41, 4.46) | 4.37 (3.70, 5.04) |  |  |  |  |
| 12 months | 3.67 (3.01, 4.32) | 3.55 (3.02, 4.09) | 3.59 (2.90, 4.28) | 0.14 (-0.66, 0.95) | 0.723 | -0.26 (-1.18, 0.67) | 0.586 |
| 24 months | 3.32 (2.61, 4.02) | 3.71 (3.08, 4.33) | 3.82 (3.08, 4.55) | 0.64 (-0.30, 1.58) | 0.178 | 0.32 (-0.68, 1.32) | 0.531 |
| **Function (mHAQ)** |  |  |  |  |  |  |  |
| Baseline | 0.53 (0.47, 0.59) | 0.59 (0.53, 0.65) | 0.59 (0.52, 0.67) |  |  |  |  |
| 12 months | 0.36 (0.29, 0.43) | 0.40 (0.34, 0.46) | 0.58 (0.50, 0.66) | -0.02 (-0.11, 0.08) | 0.753 | 0.16 (0.05, 0.27) | **0.005** |
| 24 months | 0.42 (0.34, 0.49) | 0.50 (0.43, 0.56) | 0.56 (0.47, 0.64) | 0.02 (-0.09, 0.12) | 0.730 | 0.08 (-0.05, 0.20) | 0.248 |
| **Quality of Life (EQ-5D-3L)** |  |  |  |  |  |  |  |
| Baseline | 0.70 (0.67, 0.74) | 0.67 (0.64, 0.71) | 0.60 (0.56, 0.64) |  |  |  |  |
| 12 months | 0.73 (0.69, 0.77) | 0.70 (0.67, 0.73) | 0.62 (0.58, 0.66) | 0.00 (-0.05, 0.05) | 0.993 | -0.01 (-0.00, 0.001) | 0.821 |
| 24 months | 0.70 (0.65, 0.74) | 0.67 (0.64, 0.70) | 0.62 (0.58, 0.67) | 0.00 (-0.05, 0.06) | 0.908 | 0.03 (-0.04, 0.09) | 0.408 |

Adjusted mean scores and mean differences (MD) with 95% confidence intervals (CIs) and *P*-values from linear mixed-effects regression models that include the explanatory variables BMI category, time, BMI category*time interaction term, age, gender, prednisolone dose, smoking status, and alcohol intake. *P*-values <0.05 highlighted in bold.
